# DGKζ in Glycerophospholipid Metabolism Regulates the DAG and PA Balance and Interacts With PTEN to Alleviate Brain Damage in Septic Mice With Hydrogen Inhalation: A Comparative Metabolomic and Phosphoproteomic Analysis

**DOI:** 10.1002/brb3.70761

**Published:** 2025-08-12

**Authors:** Yuanyuan Bai, Zeyu Li, Donglai Yan, Yi Jiang, Beibei Dong, Yonghao Yu

**Affiliations:** ^1^ Department of Anesthesiology Tianjin Medical University General Hospital Tianjin China; ^2^ Department of Anesthesiology, Tianjin Baodi Hospital Tianjin Medical University Baodi Hospital Tianjin China

**Keywords:** DGKζ, hydrogen, metabolomics, phosphoproteomics, sepsis

## Abstract

**Objectives:**

Therapeutic effects of hydrogen (H_2_) on sepsis‐associated encephalopathy (SAE), a severe neuroinflammatory disease, have been reported, but the underlying mechanism remains unclear. Metabolomic and phosphoproteomic analyses were utilized to explore the therapeutic mechanism of H_2_.

**Methods:**

Caecal ligation and puncture (CLP) was used to establish an animal model of sepsis, after which the animals were treated with hydrogen. Mouse brains were obtained for analysis via tandem mass tag‐based quantitative proteomics with IMAC enrichment of phosphopeptides and LC–MS/MS analysis to provide a broad overview of the metabolites. The metabolic profiles of mice in the SAE and SAE + H_2_ groups were compared by multivariate statistical analysis. Different proteins (or enzymes) were verified by western blot (WB) and immunofluorescence (IF) analyses. ELISA was used to measure the level of DAG and PA. The influence of diacylglycerol kinase ζ (DGKζ) on glycerophospholipid metabolism in the mouse hippocampus was analyzed via coimmunoprecipitation (co‐IP), and protein‒protein interactions were detected via LC‒MS/MS analysis.

**Results:**

A total of 1476 metabolites were identified, including 131 metabolic biomarkers in negative ion mode and 41 metabolic biomarkers in positive ion mode. These values were different from the standard, with variable importance for the projection (VIP) > 1 and *p* < 0.05. The correlated differential phosphoproteins found in the combined metabolomic and phosphoproteomic analyses participated in 131 pathways, and the differentially abundant metabolites were involved in 10 metabolic pathways, eight of which were related. The roles and interactions of these differentially expressed proteins and metabolites suggest that glycerophospholipid metabolism is activated in septic mice after the inhalation of hydrogen. Additionally, we quantified the downregulation of choline‐phosphate cytidylyltransferase A (Pcyt1α)/CTP/CCTα and DGKζ and the upregulation of the metabolite sn‐glycero‐3‐phosphoethanolamine in the glycerophospholipid metabolism pathway in mice in the SAE + H_2_ group compared with mice in the SAE group. The WB and IF results revealed that DGKζ expression increased in septic mice but decreased after H_2_ treatment. The ELISA showed that the expression of DAG was increased in SAE mice compared with Sham mice, while it decreased in SAE + H_2_ mice compared with SAE mice. Correspondingly, the PA level was reduced in SAE group compared with Sham group and was increased after the inhalation of H_2_. Furthermore, the regulation of DGKζ in hydrogen treatment in septic mice may be related to the interaction with phosphatase and tensin homolog (PTEN).

**Conclusion:**

H_2_ downregulates the levels of DGKζ and CCTα to alleviate brain damage in septic mice, and changes in DGKζ expression are balancing the transformation between the DAG anf PA, and it might also interact with PTEN. Thus, DGKζ may be a potential target in septic mouse therapy.

## Graphical abstract

H_2_ downregulates DGKζ and CCTα to alleviate brain damage in septic mice. The change in DGKζ has been shown to balance the transformation between DAG and PA, and it may also interact with PTEN.

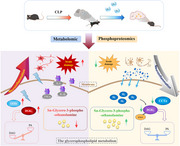

## 1 Introduction

Sepsis, defined as the presence of systemic signs of infection, results in multiple organ dysfunction syndrome (MODS) and is a vital factor in intensive care unit (ICU) patient death (Ding et al. 2018). A recent meta‐analysis in the Lancet reported that the mortality of patients with sepsis was twice that reported in previous studies (6 million people annually) (Rudd et al. 2020). As the most severe complication of sepsis, sepsis‐associated encephalopathy (SAE) leads to brain dysfunction, cognitive impairment, and even high mortality (Guo et al. 2019; Semmler et al. 2013). In addition, 16.7% of patients with SAE are confused by long‐term cognitive dysfunction (Mazeraud et al. 2020). Thus, it is necessary to determine the physiopathological mechanism of SAE to decrease its morbidity.

Known as the lightest and most widespread gas, hydrogen (H_2_) has beneficial effects on the treatment of > 170 diseases, including ischemia, metabolic syndrome, cancer, inflammation, and sepsis (Qiu et al. 2020; Saramago et al. 2019; Chen et al. 2020). Previously, we reported that H_2_ can effectively ameliorate sepsis and the sepsis‐associated dysfunction of organs such as the lung, brain, and kidney through its anti‐inflammatory, antiapoptotic, antioxidant, and neurotrophic effects (Bai, Han, et al. 2022; Bai, Li, et al. 2022; Xie et al. 2021). However, the mechanisms underlying the positive effects of H_2_ treatment on SAE are not completely understood.

With the rapid development and large‐scale application of molecular‐based technologies, multiomics techniques, including proteomics, phosphoproteomics, and metabolomics, have been used to investigate the pathophysiological and therapeutic mechanisms of various diseases (Aggarwal and Yadav 2016). Metabolomic analysis is a quantitative measurement of numerous low‐molecular‐weight molecules in particular samples, after which the data collected are converted into formats useful for data mining and informatics (Goodacre et al. 2004). Different analytical platforms have been used to investigate the potential metabolic biomarkers associated with several CNS diseases, such as Alzheimer's disease (Bogdanov et al. 2008; Ibáñez et al. 2013). Thus, based on our previous proteomic and phosphoproteomic findings in septic mice treated with hydrogen (Bai, Li, et al. 2022; Jiang et al. 2020), we further explored the protective effects of hydrogen on the brain tissues of septic mice at the metabolomic and phosphoproteomic levels to clarify the underlying therapeutic mechanism.

In this study, combined metabolomic and phosphoproteomic analyses were used to systematically determine the primary metabolic pathways, candidate genes, and signaling pathways involved in the response of septic mice to hydrogen treatment. Thus, with this combination of gene function and metabolic pathway analyses, we can improve our knowledge of the therapeutic mechanism of hydrogen treatment in septic mice, which can provide novel and effective treatment strategies for sepsis.

## 2 Materials and Methods

### 2.1 Experimental Animals

We obtained healthy adult C57BL/6J mice (males weighing 24–26 g and aged 8 weeks) from the Experimental Animal Center of the Academy of Military Sciences (Beijing, China). The animals had free access to food and water in cages in a stable environment (12:12 h light‒dark cycle; temperature, 22°C–25°C; humidity, 55% ± 10%). A total of 200 experimental animals were randomly divided into four groups: the sham, SAE, SAE + 2% hydrogen inhalation (H_2_) and SAE + hydrogen‐rich water (HW) groups. The mice in the SAE and SAE + H_2_ groups underwent caecal ligation and puncture (CLP) to establish the sepsis model, whereas the mice in the sham group underwent sham surgery. At 1 and 6 h following CLP, the mice in the SAE + H_2_ group inhaled 2% hydrogen for 60 min. The mice in the SAE + HW group were treated with 6 mL/kg hydrogen water per mouse by gavage (Zhang et al. 2014) after waking from anesthesia and beginning to move; the mice in all other groups received normal water at the same volume. The 7‐day post‐operation survival rates of the experimental mice were recorded (*n* = 20 mice per group). Tissues from the brains of 24 mice were subjected to brain phosphoproteomic and metabolomic analyses (*n* = 6 mice per group). The hippocampi of all 24 mice were subjected to western blot (WB), enzyme‐linked immunosorbent assay (ELISA), and coimmunoprecipitation (co‐IP), and tissues from another 12 experimental mice were subjected to hematoxylin and eosin (HE) and immunofluorescence staining (*n* = 3 animals per group).

### 2.2 Survival Rates of Experimental Animals

We assessed the survival rates of the experimental animals 7 days post‐operation according to previous methods (Bai, Li, et al. 2022).

### 2.3 Behavioral Experiment

The Y‐maze was used to evaluate changes in the experimental mice as described in our previous studies (Bai, Han, et al. 2022; Han et al. 2023). The contextual fear conditioning test has been widely applied in rodents to evaluate memory function and was used here as previously described (Han et al. 2023; Bouchekioua et al. 2022). (A more comprehensive account of the methodologies is provided in the Supporting Information).

### 2.4 Caecal Ligation and Puncture (CLP)

CLP was performed as previously described for the mouse SAE model (Bai, Han, et al. 2022). All experimental mice were fasted for 8 h before the operation. The experimental animals were anesthetized with isoflurane, after which their skin was sterilized. A 1‐cm incision was made in the abdomen of each mouse, the cecum was exposed, 40% of the caecum was ligated and punctured with a 21G needle, and samples of the caecal contents were removed. The caecum was returned to the abdominal cavity, and the abdominal tissues were sutured. Mice in the sham group underwent exploratory laparotomies. After all procedures, the experimental mice were injected with 1 mL of saline solution and applied lidocaine. The mice were placed on a 37°C heating blanket post‐operation.

### 2.5 Hydrogen Treatment

H_2_ was administered as described in our previous study (Bai, Han, et al. 2022). The experimental mice were placed into a closed resin box with an inlet and an outlet. H_2_ was produced by a TF‐1 gas flowmeter (4 L/min; YUTAKA Engineering Corp.). A detector (HY‐ALERTA Handheld Detector Model 500; H_2_ Scan) was used to measure the H_2_ concentration. The exhaled CO_2_ was absorbed by Baralyme. The mice in the SAE + H_2_ group inhaled H_2_ gas for 60 min starting 1‐ and 6‐h post‐operation, while the mice in the other groups inhaled the room air.

### 2.6 Hydrogen‐Rich Water Treatment

Hydrogen was generated and administered to the experimental mice as described previously (Han et al. 2023; Lian et al. 2021). The main steps in generating HW are to dissolve hydrogen in normal water under high pressure (0.4 MPa) for 4 h and then store it under atmospheric pressure at 4°C. The HW was freshly prepared, and its concentration was measured using a hydrogen electrode.

### 2.7 Antibodies and Reagents

The following primary antibodies were used: anti‐diacylglycerol kinase ζ (DGKζ) (Cat# ab239080, Abcam, Britain and Cat# ab239081, Abcam, Britain), anti‐PTEN (Cat# 9559, Cell Signaling Technology, USA), and mouse anti‐GAPDH (Cat# DF6226, Affinity, USA); secondary antibodies: goat anti‐mouse (Cat# 31430, Invitrogen, USA), goat anti‐rabbit (Cat# 31466, Invitrogen, USA), and rabbit IgG control polyclonal (Cat# 30000‐0‐AP, Proteintech, USA). Coomassie blue fast staining solution (Cat# P0017, Beyotime, China) was also used. Secondary fluorophore‐bound IgGs (Alexa Fluor series) for immunofluorescence analyses were obtained from Life Technologies.

### 2.8 HE Staining

At 24‐h post‐operation, the experimental animals were sacrificed, and their brains were removed for HE staining. The experimental animals were transcardially perfused with phosphate‐buffered saline followed by 4% paraformaldehyde. The brain tissues were excised, fixed, embedded, and subsequently cut into sections. After deparaffinization and dehydration, the samples were stained with HE. Regional neuropathological changes were observed with a BX51 microscope (Olympus, Tokyo, Japan).

### 2.9 ELISA

The brain tissues of the experimental animals were homogenized and centrifuged (10,000 × *g*, 4°C), and the supernatant was collected. The expressions of IL‐1β, TNF‐α, IL‐6 (Cat# YJ098416, Cat# YJ002095, and Cat# YJ063159, respectively; Mlbio, China) and DAG (Cat# BY‐JZF0298, BYabscience, China) and PA (Cat# YJ432589, Mlbio, China) were evaluated using ELISA kits.

### 2.10 Western Blot

Twenty‐four hours post‐operation, total protein was extracted from the hippocampal tissues of mice from the different experimental groups to verify the expression of DGKζ and PTEN as described in our previous study (Bai, Han, et al. 2022; Bai, Li, et al. 2022). Briefly, after the total protein content of the samples was measured, the samples were boiled and denatured. The proteins were subsequently separated via SDS‒PAGE and transferred to a polyvinylidene fluoride membrane. After being blocked with primary antibodies (DGKζ: 1:1000; PTEN: 1:1000; GAPDH: 1:5000) and secondary antibodies (1:5000), the bands were visualized with ECL detection reagents. The protein bands were analyzed with ImageJ software, and the levels of the target proteins were normalized to those of GAPDH. The process was repeated six times.

### 2.11 ImmunoFluorescence Staining

The above‐collected brain tissues were sliced and prepared as described for HE staining. The slices were incubated with primary antibody (DGKζ: 1:200) overnight at 4°C in PBS supplemented with 1% BSA. The slices were subsequently washed with PBS three times for 5 min each, after which they were incubated with fluorescent secondary antibodies at room temperature for 1 h. After washing, the nuclei were stained with DAPI. The results were observed via fluorescence microscopy (Olympus).

### 2.12 Coimmunoprecipitation

The samples used in the experiments were prepared according to the instructions for the Beaver Beads Protein A/G Matrix Immunoprecipitation Kit (Beaver Nano‐Technologies Co., China). After centrifugation, the protein concentrations of the supernatants were measured via the bicinchoninic acid (BCA) assay. The extracts (0.500 mg of protein) from each group were incubated with 5 µg of a rabbit monoclonal anti‐DGKζ antibody (Cat# ab239080; Abcam, Britain) and anti‐PTEN (Cat# 9559, Cell Signaling Technology) or nonspecific IgG (5 µg) and 50 mL of immunomagnetic beads overnight at 4°C with gentle agitation. The precipitates were washed four times with lysis buffer, denatured with SDS sample buffer, and then quantified via WB analysis.

### 2.13 Methods of Metabolomic and Phosphoproteomic Analyses

Details of the methods used for the metabolomic and phosphoproteomic analyses are provided in the Supporting Information.

### 2.14 Combined Analysis of the Metabolomics and Phosphoproteomics Data

#### 2.14.1 Principal Component Analysis (PCA)

PCA was implemented with SIMCA version 14.1 using quantitative data from the two omics analyses.

#### 2.14.2 KEGG Pathway Analysis and Expression Profiling of the Differentially Modified Peptides in the Significantly Enriched KEGG Pathways

All differentially expressed proteins/modified peptides/genes and metabolites were queried and mapped to pathways via the online Kyoto Encyclopedia of Genes and Genomes (KEGG, http://www.kegg.jp/). The combination of KEGG annotation and enrichment results from the two omics datasets and the corresponding graphs, including the Venn diagram, bar plot, and heatmap, were generated with R version 3.5.1. *Z* score normalization was implemented on the quantitative data of the target differentially modified peptides.

#### 2.14.3 Expression Profiles of the Differentially Modified Peptides in Shared Significant Enrichment Pathway in the KEGG Pathway

Z‐score normalization was performed on the quantitative data of the target differentially modified peptides, then heatmap was drawn with R Version 3.5.1 (Distance Matrix Computation: Euclidean, Hierarchical Clustering: complete linkage).

#### 2.14.4 Correlation

The differentially abundant proteins/modified peptides/genes/metabolites/lipids were log2 scaled (TMT/iTRAQ) or Z score scaled (label‐free) and concatenated into one matrix. The correlation coefficients among all the molecules in the matrix were subsequently calculated via the Pearson algorithm in R version 3.5.1.

#### 2.14.5 Correlation Network

Pearson correlation coefficients among the differentially expressed proteins/modified peptides/genes and metabolites/lipids were loaded into Cytoscape version 3.5.1, and a correlation network was constructed.

#### 2.14.6 Multivariate Data Analysis

MVDA was performed on differentially expressed abundant proteins/modified peptides/genes and metabolites with SIMCA version 14.1.

### 2.15 Statistical Analysis

After sum normalization, the processed data were analyzed via the R package ropls, where they were subjected to multivariate data analyses. Sevenfold cross‐validation and response permutation testing were applied to assess the robustness of the model. Student's *t* test was used to identify the significance of differences between two groups of independent samples. VIP > 1 and *p* < 0.05 were used to screen for significantly changed metabolites. Pearson's correlation analysis was applied to determine the correlation between two variables. GraphPad Prism software (version 8.0) was used. The survival rates were analyzed via the log‐rank test. The data are presented as the means ± standard deviations. Two‐way ANOVA with repeated measures was used to analyze the differences between treatments and groups, followed by Tukey's multiple comparisons test. *p* < 0.05 was considered statistically significant.

## 3 Results

### 3.1 Survival Rate, Brain Damage, and Cognitive Dysfunction in Septic Mice

**FIGURE 1:**
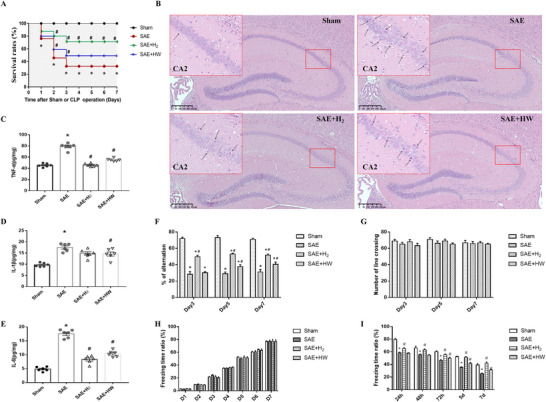
Effects of molecular hydrogen (H_2_ and HW) treatment on sham‐ or CLP‐induced septic mice. (A) Survival rate (*n* = 20 mice per group); (B) HE staining (*n* = 3 mice per group); (C) TNF‐α, (D) IL‐1β, and (E) IL‐6 levels in the hippocampus were measured by ELISA (*n* = 6 mice per group). The cognitive function of the mice was assessed by the Y‐maze test, which included performing the spontaneous alternation test (F) and counting the number of line crossings (G), and by the contextual fear conditioning test, which included determining the training time‒freezing time ratio (H) and the evaluation stage (I). The data are presented as the means ± SDs. *p < 0.05 versus the sham group. #*p* < 0.05 versus the SAE group. HW: hydrogen‐rich water; CLP: caecal ligation and puncture. TNF‐α: tumor necrosis factor α; IL: interleukin.

Post‐operation physiological and pathological changes such as unkempt hair, loose stools, reduced activity, apathy, astasia, anorexia, and hypothermia in the first 24 h were compared between experimental groups (Jiang et al. 2020). At 7 days post‐operation, all of the mice in the sham group and significantly fewer in the other three groups had (*p* < 0.05). However, the survival rates among the septic mice treated with hydrogen (70% of mice in the SAE + H_2_ group and 49% of mice in the SAE + HW group) were significantly greater than that of the SAE mice (35% of mice in the SAE group; *p* < 0.05; Figure 1A).

HE staining revealed that the pyramidal neuron structure of the hippocampal CA2 region, which is a key component for memory, was clear in mice in the sham group but disrupted and disordered in mice in the SAE group (*p* < 0.05). In addition, neuronal damage was alleviated in mice in the SAE + H_2_ and SAE + HW groups compared with those in the SAE group, and there was no significant difference in pyramidal neuron structure between mice in the SAE + H_2_ and SAE + HW groups (*p* > 0.05; Figure 1B).

Interleukin (IL)‐1β, tumor necrosis factor (TNFα), and IL‐6 are the most vital cytokines that promote the inflammatory response in sepsis patients and are key biomarkers of septic shock in systemic inflammatory response syndrome patients (Grondman et al. 2020). To investigate the effect of hydrogen on the inflammatory reaction in brain tissues, we measured the levels of TNF‐α, IL‐1β, and IL‐6 at 24 h to determine the severity of the injury. Mice in the SAE + H_2_ and SAE + HW groups showed lower TNF‐α, IL‐1β, and IL‐6 levels than in the SAE group of mice (*p* < 0.05; Figures 1C–E).

The Y‐maze spontaneous alternation test and fear conditioning test were used to evaluate the memory and learning ability and the environmental correlation of fear condition memory of the mice, respectively. The percentage of alternations in the Y‐maze at 3, 5, and 7 days post‐operation was lower in septic mice (those in the SAE, SAE + H_2_, and SAE + HW groups) than in mice in the sham group. While the percentage of alternations was greater in mice in the SAE + H_2_ group than in those in the SAE and SAE + HW groups (Figure 1F; *p* <0.05), at 5 and 7 days post‐operation, this percentage was greater in mice in the SAE + HW group than in those in the SAE group (*p* <0.05). The number of line crossings was not significantly different among the groups (Figure 1G; *p* > 0.05). The fear conditioning test revealed that the percentage of freezing time was greater in mice in the SAE + H_2_ group than in those in the SAE and SAE + HW groups, but at 5 and 7 days post‐operation, this percentage was greater in mice in the SAE + HW group than in those in the SAE group (*p* < 0.05; Figures 1H,I).

Overall, we found that hydrogen therapy (H_2_ or HW) effectively alleviated brain damage and cognitive dysfunction in septic mice and improved their survival rate.

### 3.2 Metabolomic Analysis of Brain Tissues From Septic Mice

**FIGURE 2:**
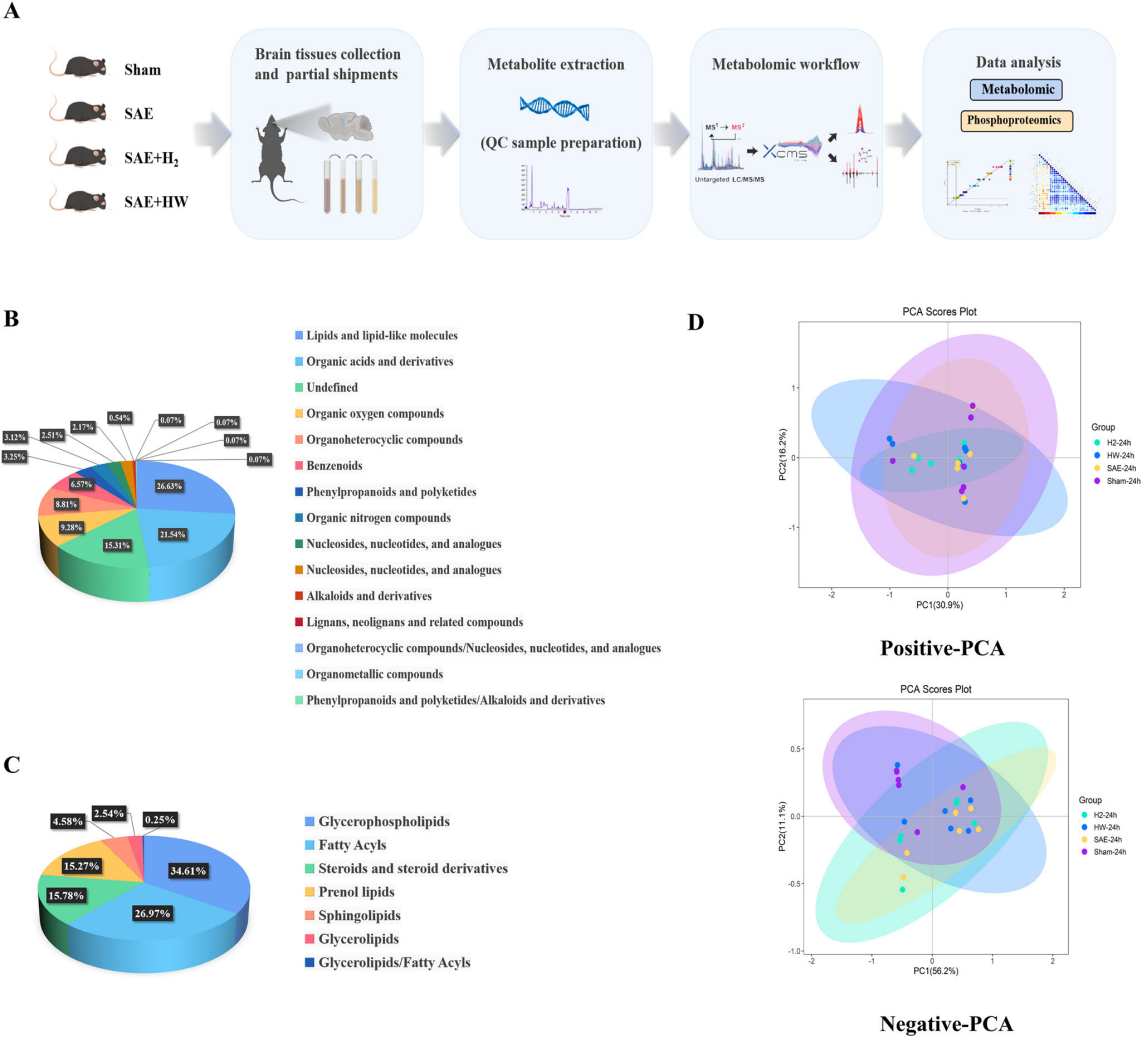
Identification and characterization of metabolites in the brain tissues of septic mice treated with hydrogen (H_2_ and HW). (A) Experimental workflow for metabolomic and phosphoproteomic analyses of brain tissues from mice in the sham, SAE, SAE + H_2_ and SAE + HW groups (*n* = 6 mice per group). (B) Pie chart of the superclasses of chemical taxonomy in the comparison among mice in the four groups. (C) Pie chart of the further classification of lipids and lipid‐like molecules. (D) The positive and negative PCA results revealed differences among the four groups. SAE: sepsis‐associated encephalopathy; PCA: principal component analysis.

The brain tissues samples collected from mice in the four groups (sham, SAE, SAE + H_2_, and SAE + HW) (*n* = 6) were also subjected to metabolomic analysis (Figure 2A). A total of 1476 metabolites were identified, including 852 and 624 metabolites in positive ion mode and negative ion mode, respectively. Among these, 26.63% were lipid and lipid‐like molecules; 21.55% were organic acids and derivatives; 9.28% were organic oxygen compounds; 8.81% were organoheterocyclic compounds; 6.57% were benzenoids; 3.25% were phenylpropanoids and polyketides; 3.12% were organic nitrogen compounds; and 2.51% were nucleosides, nucleotides, and analogues (Figure 2B). Furthermore, the lipid and lipid‐like molecules were categorized into glycerophospholipids (34.61%), fatty acyls (26.97%), steroids and steroid derivatives (15.78%), prenol lipids (15.27%), sphingolipids (4.58%), glycerolipids (2.54%), and others (0.25%) (Figure 2C). PCA revealed that hydrogen treatment was vital for septic mice, and the differences among mice in the SAE, SAE + H_2_, and SAE + HW groups were significant (Figure 2D). To analyze the expression patterns of all qualitative metabolites in each group, the changes in metabolite expression were characterized.

### 3.3 Differentially Abundant Metabolites and Correlated KEGG Pathway Analysis

**FIGURE 3:**
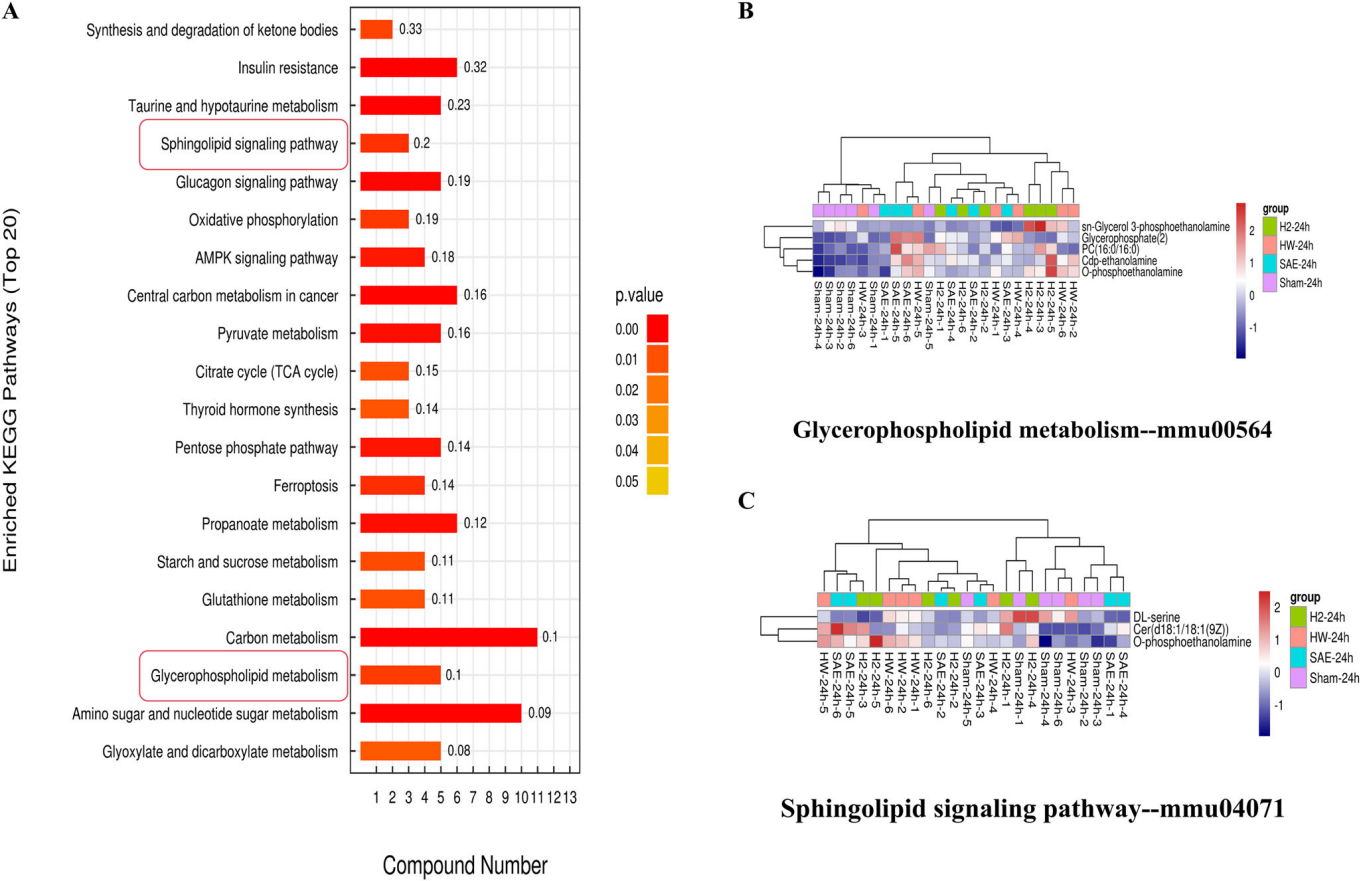
KEGG enrichment pathways of the differentially expressed correlated metabolites. (A) The top 20 enriched KEGG pathways are listed (FDR < 0.05). The *X*‐axis indicates the number of proteins, with the corresponding KEGG pathway marked on the *y*‐axis. (B) Glycerophospholipid metabolism enrichment and (C) sphingolipid signaling pathway enrichment of some significant metabolites.

Based on the parameters of VIP >1 and *p* < 0.05, we identified the differentially abundant metabolites in the brain tissues of septic mice. There were 131 metabolic biomarkers in negative ion mode and 41 metabolic biomarkers in positive ion mode whose levels differed among all group of mice. In addition, we performed pathway analysis to elucidate the biological roles of the differentially abundant metabolites and the signaling events in which they participate. KEGG enrichment pathway analysis with Fisher's exact test (FDR < 0.05) revealed that the top 20 pathways were related to insulin resistance, carbon metabolism, the glucagon signaling pathway, ferroptosis, the sphingolipid signaling pathway, and glycerophospholipid metabolism, as shown in Figure 3A and Table 1. Glycerophospholipid metabolism enrichment was associated with sn‐glycero‐3‐phosphoethanolamine, glycerophosphate (2), PC (16:0/16:0), CDP‐ethanolamine, and O‐phosphoethanolamine (Figure 3B). The sphingolipid signaling pathway was related to the differentially abundant metabolites DL‐serine, Cer(d18:1/18:1(9Z)), and O‐phosphoethanolamine (Figure 3C). The levels of all the metabolites involved in glycerophospholipid metabolism and sphingolipid metabolism significantly changed, indicating that these pathways are closely related to the mechanism underlying hydrogen treatment in septic mice.

**TABLE 1:** Top 20 significant KEGG enrichment pathway related with the differential metabolites in the mice brain tissues.

Pathway_Hierarchy1	Pathway_Hierarchy2	Map_ID	Map_Name	Test	TestAll	Ref	RefAll	Test_per	Ref_per	Over_Under	*p* value	FDR	Rich factor
Human diseases	Endocrine and metabolic disease	mmu04931	Insulin resistance	6	93	19	4160	6.451613	0.456731	Over	2.27904E‐06	0.000252974	0.315789474
Metabolism	Global and overview maps	mmu01200	Carbon metabolism	11	93	114	4160	11.827957	2.740385	Over	3.70937E‐05	0.002058703	0.096491228
Metabolism	Metabolism of other amino acids	mmu00430	Taurine and hypotaurine metabolism	5	93	22	4160	5.376344	0.528846	Over	9.77032E‐05	0.003133127	0.227272727
Metabolism	Carbohydrate metabolism	mmu00520	Amino sugar and nucleotide sugar metabolism	10	93	108	4160	10.752688	2.596154	Over	0.000121916	0.003133127	0.092592593
Human diseases	Cancer: overview	mmu05230	Central carbon metabolism in cancer	6	93	37	4160	6.451613	0.889423	Over	0.000141132	0.003133127	0.162162162
Organismal systems	Endocrine system	mmu04922	Glucagon signaling pathway	5	93	26	4160	5.376344	0.625	Over	0.000227374	0.004206426	0.192307692
Metabolism	Carbohydrate metabolism	mmu00620	Pyruvate metabolism	5	93	31	4160	5.376344	0.745192	Over	0.000537652	0.00848138	0.161290323
Metabolism	Carbohydrate metabolism	mmu00640	Propanoate metabolism	6	93	48	4160	6.451613	1.153846	Over	0.000611271	0.00848138	0.125
Metabolism	Carbohydrate metabolism	mmu00030	Pentose phosphate pathway	5	93	35	4160	5.376344	0.841346	Over	0.000957257	0.011806171	0.142857143
Environmental information processing	Signal transduction	mmu04152	AMPK signaling pathway	4	93	22	4160	4.301075	0.528846	Over	0.001257673	0.013960173	0.181818182
Cellular processes	Cell growth and death	mmu04216	Ferroptosis	4	93	29	4160	4.301075	0.697115	Over	0.003623488	0.036564283	0.137931034
Environmental information processing	Signal transduction	mmu04071	**Sphingolipid signaling pathway**	3	93	15	4160	3.225806	0.360577	Over	0.004050205	0.037464394	0.2
Metabolism	Energy metabolism	mmu00190	Oxidative phosphorylation	3	93	16	4160	3.225806	0.384615	Over	0.004904834	0.04187974	0.1875
Metabolism	Lipid metabolism	mmu00564	**Glycerophospholipid metabolism**	5	93	52	4160	5.376344	1.25	Over	0.00568524	0.045075832	0.096153846
Metabolism	Lipid metabolism	mmu00072	Synthesis and degradation of ketone bodies	2	93	6	4160	2.150538	0.144231	Over	0.006995386	0.051765859	0.333333333
Metabolism	Carbohydrate metabolism	mmu00500	Starch and sucrose metabolism	4	93	37	4160	4.301075	0.889423	Over	0.008795053	0.05959836	0.108108108
Metabolism	Carbohydrate metabolism	mmu00020	Citrate cycle (TCA cycle)	3	93	20	4160	3.225806	0.480769	Over	0.00936039	0.05959836	0.15
Metabolism	Metabolism of other amino acids	mmu00480	Glutathione metabolism	4	93	38	4160	4.301075	0.913462	Over	0.009664599	0.05959836	0.105263158
Organismal systems	Endocrine system	mmu04918	Thyroid hormone synthesis	3	93	21	4160	3.225806	0.504808	Over	0.010745994	0.062779228	0.142857143
Metabolism	Carbohydrate metabolism	mmu00630	Glyoxylate and dicarboxylate metabolism	5	93	62	4160	5.376344	1.490385	Over	0.011887034	0.064719078	0.080645161

The bold values (sphingolipid signaling pathway and Glycerophospholipid metabolism)in table 1 was correspond to the boxed pathways in Figure 3A and serve as a transition to introduce the explanations provided in Figures 3B and 3C, as described in the line 8–11 of 3.3 section.

### 3.4 Combined Metabolomic and Phosphoproteomic Analyses

**FIGURE 4:**
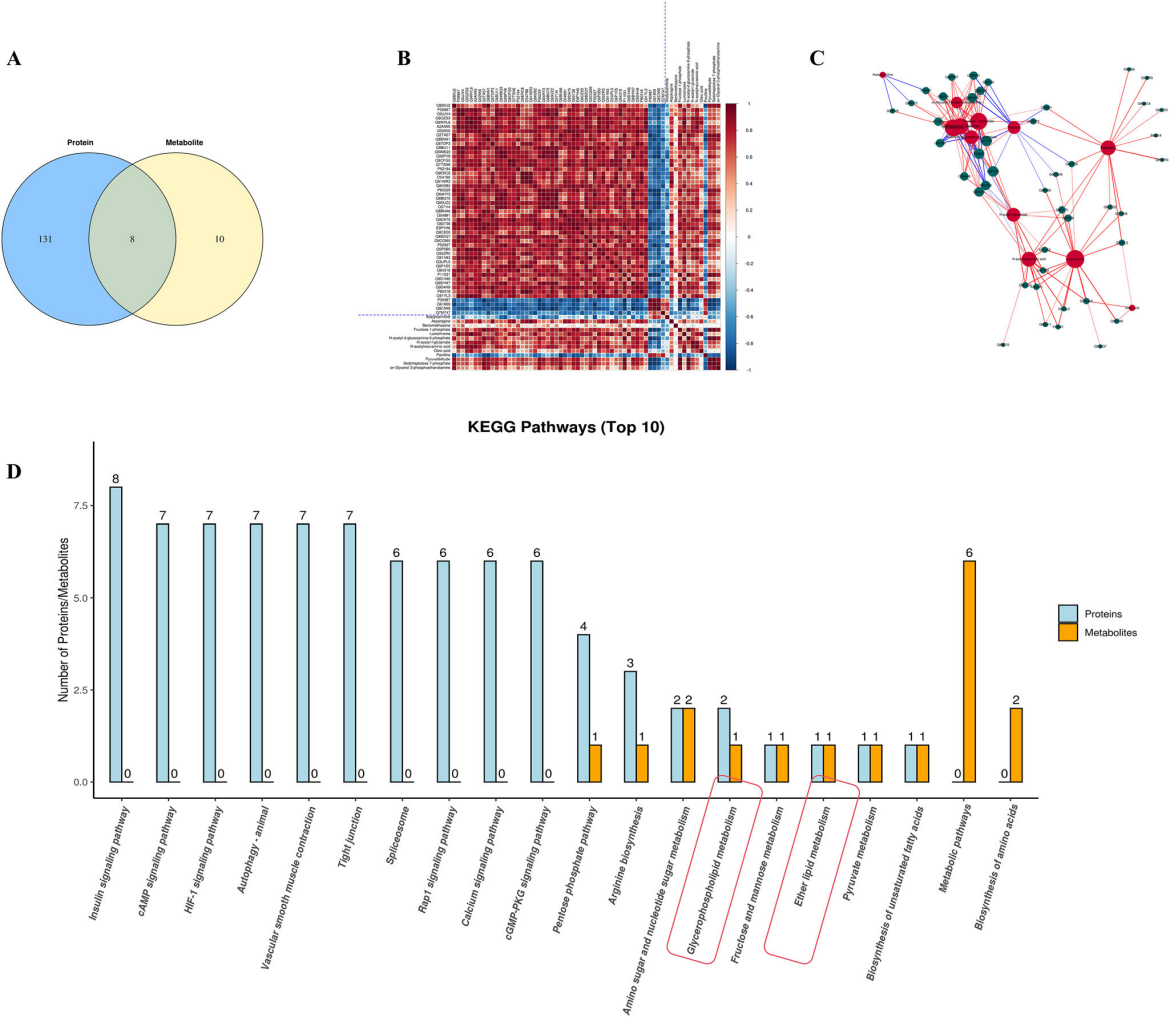
Combined metabolomic and phosphoproteomic analyses of mice in the SAE and SAE + H_2_ groups. (A) Eight metabolic pathways were identified from the comparison between the two groups. (B) The correlation coefficients (*r*) between the significantly different proteins and the significantly different metabolites were calculated, and the correlations are presented in the form of a correlation coefficient matrix heatmap. (C) The correlation network (*r* ≥ 0.5 and *p* < 0.05) of the 8 metabolic pathways is shown. (D) The top 10 KEGG pathways included glycerophospholipid metabolism and ether lipid metabolism.

To further investigate the therapeutic mechanism of hydrogen treatment in septic mice, we carried out combined metabolomic and modified protein (phosphoproteomic) analyses. As shown in Figure 4A, the differential phosphoproteins participated in 131 pathways, and the differentially abundant metabolites were involved in 10 metabolic pathways, eight of which were related. Based on the Pearson correlation analysis, the correlation coefficients (*r*) between the significantly different proteins and the significantly different metabolites were calculated, and the correlations are presented in the form of a correlation coefficient matrix heatmap (Figure 4B). In addition, the correlation network (*r* ≥ 0.5 and *p* < 0.05) of the eight metabolic pathways is shown in Figure 4C. The top 10 KEGG pathways, which included the greatest number of differentially expressed proteins and metabolites, included the pentose phosphate pathway, arginine biosynthesis, amino sugar and nucleotide sugar metabolism, biosynthesis of unsaturated fatty acids, ether lipid metabolism, glycerophospholipid metabolism, fructose and mannose metabolism, and pyruvate metabolism (Figure 4D). Thus, we suggest that glycerophospholipid metabolism was activated in mice in the SAE + H_2_ group compared with those in the SAE group and played a vital role in the therapeutic effects of hydrogen.

**FIGURE 5:**
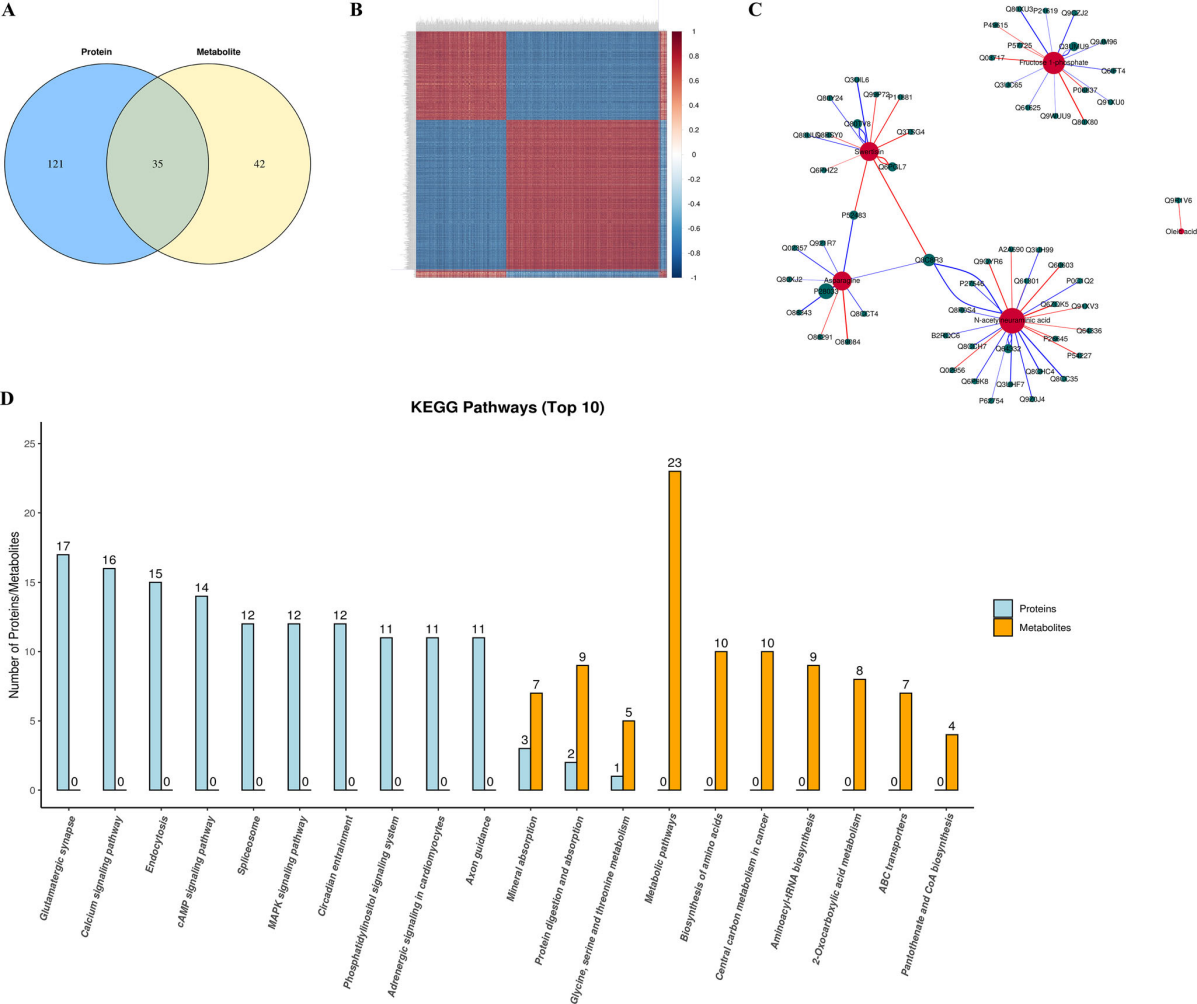
Combined metabolomic and phosphoproteomic analyses of mice in the SAE and SAE + HW groups. (A) Thirty‐five common pathways were identified from the comparison between the two groups. (B) Heatmap and (C) network graph showing the correlations between the two groups via Pearson correlation analysis. (D) The top 10 KEGG pathways containing the maximum number of differentially expressed proteins and metabolites are shown.

By comparing the SAE and SAE + HW groups, we found 121 pathways correlated with the different phosphoproteins and 42 pathways associated with the different metabolites, of which 35 metabolic pathways were shared (Figure 5A). A heatmap and network graph were used to show the correlation between the two groups via Pearson correlation analysis (Figure 5B,C). Furthermore, the top 10 KEGG pathways containing the greatest number of differentially expressed proteins and metabolites were involved in mineral absorption, protein digestion and absorption, and glycine, serine, and threonine metabolism (Figure 5D).

### 3.5 Changes in Glycerophospholipid Metabolism in Mice in the SAE + H_2_ Group

**FIGURE 6:**
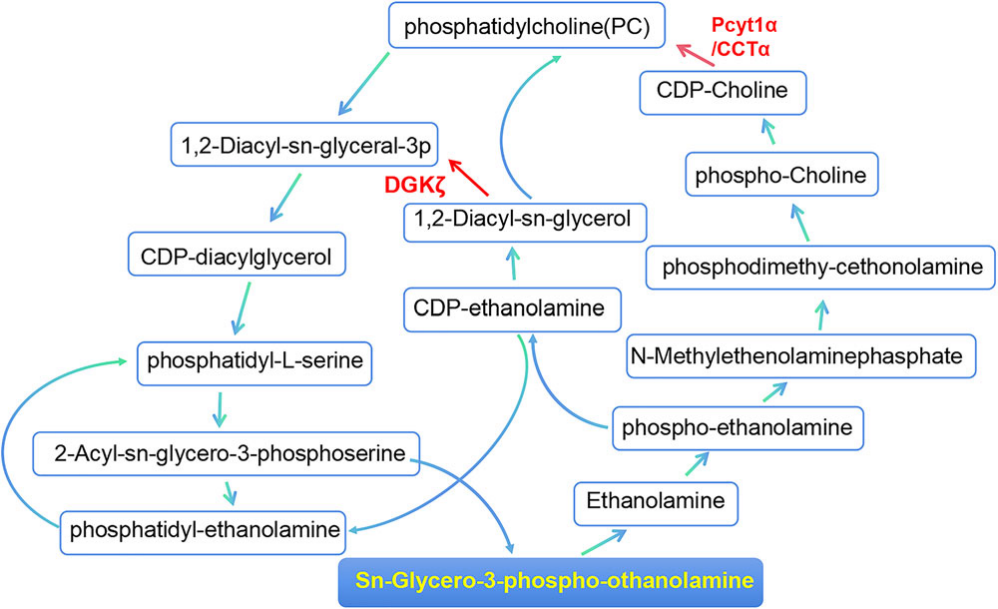
Two phosphoproteins involved in glycerophospholipid metabolism (DGKζ and Pcyt1α/CCTα) were differentially expressed and were downregulated in mice in the SAE + H_2_ group compared with mice in the SAE group, and one metabolite (sn‐glycero‐3‐phosphoethanolamine) was upregulated in mice in the SAE + H_2_ group compared with mice in the SAE group. DGKζ: diacylglycerol kinase ζ; Pcyt1α/CCTα: choline‐phosphate cytidylyltransferase A/phosphocholine cytidylyltransferase.

The roles and interactions of these differentially expressed proteins and metabolites suggest that glycerophospholipid metabolism was activated in septic mice after hydrogen inhalation (Figure 6). Additionally, we detected the downregulation of choline‐phosphate cytidylyltransferase A (Pcyt1α)/CTP/CCTα(2.7.7.15) and DGKζ (2.7.1.107) and the upregulation of sn‐glycero‐3‐phosphoethanolamine in the glycerophospholipid metabolism pathway based on the analysis of the differentially expressed proteins. Located prominently in cell membranes, phospholipids play an important role in cell division by supplying membrane components. In addition, the induction of phosphatidylcholine (PtdCho) biosynthesis is important for cell proliferation (Cui et al. 1996).

Pcyt1α/CCTα, the key rate‐limiting enzyme located in the nucleus, is involved in PtdCho biosynthesis (Li and Vance 2008). There is evidence that Pcyt1α/CCTα may be a promising biomarker for some cancers, including lung, head, and neck squamous cell carcinomas (Yang et al. 2020). Additionally, Pcyt1α/CCTα participates in the formation of the nucleoplasmic reticulum and the phospholipid bilayer of the nuclear membrane and is crucial for the anchorage‐independent growth of Ras‐transformed cells, apoptosis resistance, cell proliferation, and synthesis of PtdCho in cancers (Arsenault et al. 2013; Linkous et al. 2010). Interestingly, we found that hydrogen alleviated sepsis‐induced neuroinflammation via the mTOR‐dependent autophagy pathway (Zhuang et al. 2020). This finding is consistent with the hydrogen‐induced downregulation of Pcyt1α in septic mice, alleviating brain damage and cognitive impairment in this study.

Because of their diverse expression patterns, tissue distributions, and biological functions, DGKs play key roles in malignant tumors (Torres‐Ayuso et al. 2014). In addition, dysfunction of DGKs, which regulate the balance of diacylglycerol (DAG) and phosphatidic acid (PA), might contribute to a variety of diseases, including biodisorders and diabetes (Kakefuda et al. 2010). Because the myristoylated alanine‐rich C‐kinase substrate (MARCKS) domain regulates the nuclear localization of DGKζ (Topham et al. 1998), type IV DGKζ might have diverse functions in different cellular events, including the regulation of mTORC1 and lipid metabolism in human colorectal tumor cells through SREBP‐1 (Torres‐Ayuso et al. 2015) and the regulation of the crosstalk between NF‐κB and the p53 signaling pathway, which are involved in cell survival and death (Tanaka et al. 2013). Furthermore, DGKζ might play a crucial role in signaling pathways related to PIP2 or PI3K, such as the PI3K/AKT/mTOR pathway, which regulates many intracellular upstream signaling pathways involved in cell growth, metabolism, angiogenesis, and vesicle trafficking (Gu et al. 2019). Moreover, we found that sepsis‐induced neuroinflammation was attenuated by molecular hydrogen, dependent on the mTOR‐autophagy‐dependent pathway (Zhuang et al. 2020).

### 3.6 The Regulation of DGKζ in Septic Mice Treated With H_2_ and the Interaction of DGKζ With PTEN

**FIGURE 7:**
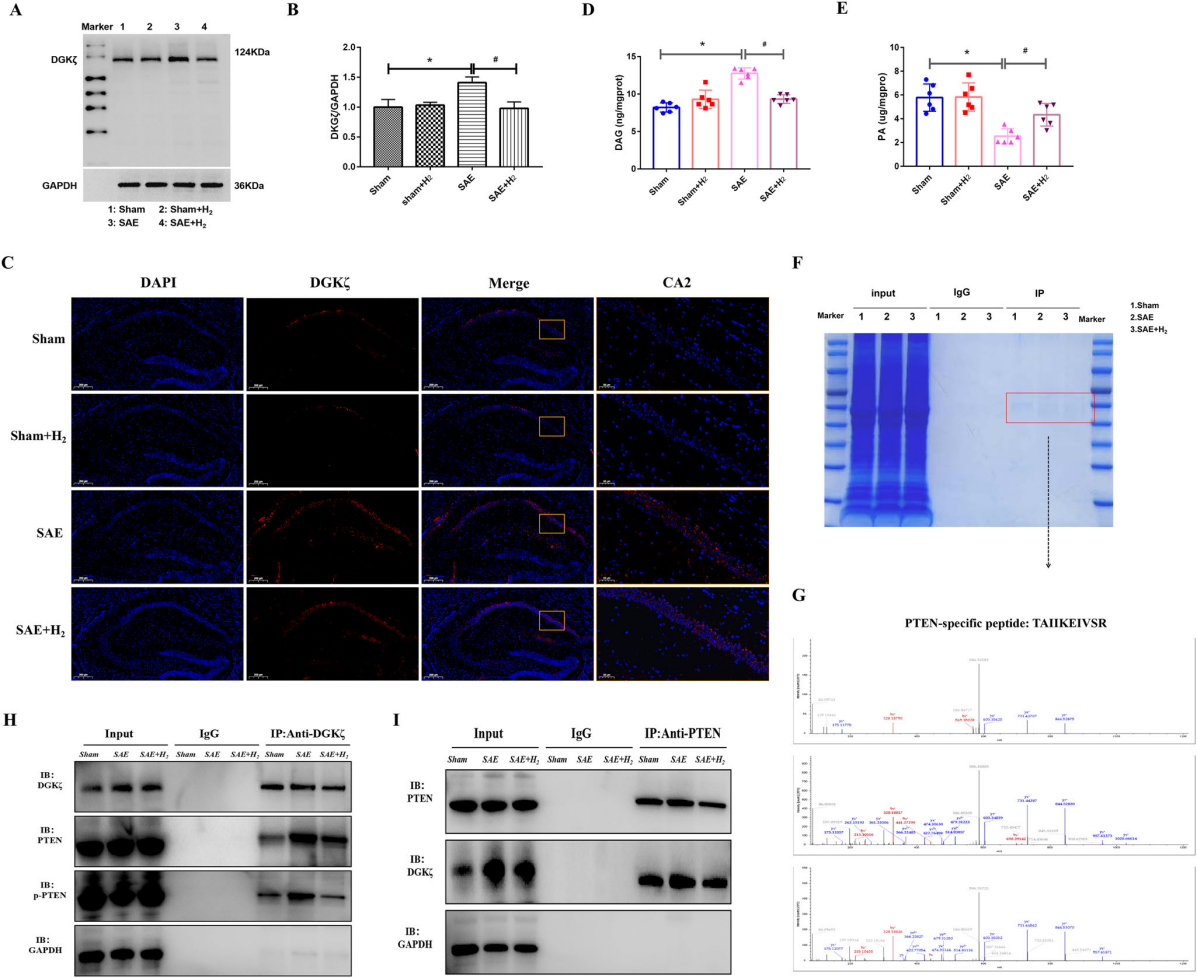
Effect of DGKζ in septic mice treated with inhaled hydrogen and the interaction of DGKζ with proteins. (A) and (B) Western blot of DGKζ in the hippocampal tissues of septic mice 24‐h post‐operation (*n* = 6 mice per group); GAPDH was used as a loading control. The data are presented as the means ± SDs; *p < 0.05, compared with the sham group; #*p* < 0.05, compared with the SAE group. (C) Immunofluorescence images showing the expression of DGKζ (*n* = 3 mice per group). (D) and (E) DAG and PA expression level in hippocampal tissues of septic mice were measured by ELISA (*n* = 6 mice per group); The data are presented as the means ± SDs; *p < 0.05, compared with the sham group; #*p* < 0.05, compared with the SAE group. (F) Coomassie blue staining and (G) the fragment mass spectra analysis of the hippocampal tissues of septic mice revealed successful IP results. (H) and (I) Co‐IP results showing the PPIs between DGKζ and PTEN in septic mice (*n* = 6 mice per group). IP: immunoprecipitation; PPI: protein–protein interaction.

With western blotting, we showed that the expression of DGKζ increased in septic mice but decreased after H_2_ treatment (Figures 7A,B). The immunofluorescence results revealed the same changes in gene expression in the hippocampus, including in the CA2 region, in mice in the four experimental groups (Figure 7C). As previously mentioned, DGKζ is primarily localized in the nuclei of various types of cells, where it catalyzes the conversion of DAG to PA, thereby regulating DAG‐related signaling pathways and PA‐related signaling pathways (Kakefuda et al. 2010). Interestingly, we found that changes in DGKζ in sepsis mice led to an imbalance in the DAG and PA signaling pathways. The level of DAG of SAE mice was increased, while it was reduced in SAE mice treated with hydrogen inhalation. Conversely, the levels of PA were significantly decreased in SAE mice but increased in the SAE + H_2_ group (Figure 7D,E).

To identify other potential pathways, we adopted the following methods. First, we used an anti‐DGKζ antibody to co‐IP the DGKζ‐binding proteins in the hippocampi of mice in the sham, SAE, and SAE + H_2_ groups. WB analysis revealed that DGKζ was markedly immunoprecipitated (Figure 7H), suggesting that the IP assay was successful. The resulting IP products were subsequently subjected to SDS‐polyacrylamide gel electrophoresis (PAGE) and MS analysis. Coomassie blue staining and fragment mass spectra analysis confirmed the presence of the peptides from mice in the three septic groups (SAE, SAE + H_2_, and SAE + HW; Figure 7F,G). Consistently, the commercial PTEN antibody recognized PTEN in the anti‐DGKζ IP (Figure 7H). It was also found that DGKζ was detected in the anti‐PTEN IP (Figure 7I).

These results indicate that hydrogen regulates the expression of DGKζ and balance the DAG transfer to PA and its related pathways, thereby changing the cellular physiology, including energy flow, signaling balance, and phospholipid metabolism in H_2_‐treated septic mice. In our previous study, we demonstrated that hydrogen can alleviate brain damage and cognitive impairment in septic mice by regulating the PI3K/AKT/mTOR signaling pathway, which plays a key function in cell proliferation, growth, and development (Bai, Li, et al. 2022; Wang et al. 2025). It has been known that DGKζ catalyzes the conversion of DAG to PA, a key step in lipid metabolism, and PA modulates lipid kinases like PI3K, impacting the PI3K/AKT signaling pathway (Sakane et al. 2025). It has also been suggested that DGKζ knockdown suppresses cell proliferation and survival, which is likely attributed to its effects on lipid metabolism and the PI3K/AKT pathway (K. Liu et al. 2021; Y. Liu et al. 2024). PTEN, which is a lipid and protein phosphatase, dephosphorylates phosphatidylinositol‐3,4,5‐trisphosphate (PIP3), a key PI3K downstream product, blocking AKT activation and negatively regulating the PI3K/AKT pathway (Endicott and Miller 2024; Tufail 2025). The lipid metabolism of DGKζ regulation may indirectly affect PTEN function by changing PI3K/AKT lipid metabolite levels. Alternatively, the declines of the DGKζ activity may lead to the falling levels of PA, which may reduce PI3K activity and AKT phosphorylation. It could boost PTEN activity or expression as a compensatory mechanism (Lee et al. 2018). Because phospholipase C (PLC) hydrolyzes PIP2 (PIP3's precursor) to produce DAG and IP3, while phospholipase D (PLD) mainly hydrolyzes phosphatidylcholine (PC) to generate PA and choline, PLC and PLD can indirectly influence DGKζ (Cocco et al. 2015; Ubeysinghe et al. 2023; Kanemaru and Nakamura 2023; Tariq and Luikart 2021). PTEN can adjust the activities of PLD and PLC by regulating PIP3, and it may also affect DGKζ indirectly (Naik 2010). In septic mice models, hydrogen gas activates the PI3K/AKT pathway, which may have a negative feedback effect on PTEN, thus reducing PTEN‐mediated dephosphorylation of PIP3. Consequently, this mechanism might indirectly upregulates the activities of PLD and PLC, leading to an increase in PA production. The elevated levels of PA may subsequently exert negative feedback regulation on DGKζ expression, modulating its role in cellular signaling and lipid metabolism within the context of sepsis.

## 4 Discussion

SAE, regarded as the most serious complication caused by sepsis, involves blood–brain barrier disruption, cognitive dysfunction, brain damage, behavioral defects, and neurological inflammation (Barichello et al. 2005; He et al. 2023). Thus, potential treatment agents and targets for SAE have been explored by researchers. CLP is recognized as a classic model of sepsis in animals (Calsavara et al. 2015). After successfully performing CLP surgery in septic mice, we combined analyses of the brain histopathology and inflammatory response and neurobehavioral dysfunction to establish the mouse model of SAE used in this study.

Hydrogen can be administered in different ways and has therapeutic effects on different diseases through antioxidative, anti‐inflammatory, antiapoptotic, antishock, and autophagy regulatory effects (Qi et al. 2021), especially for treating sepsis. We investigated the therapeutic effects of H_2_ on sepsis (specifically SAE) and further investigated the underlying mechanisms. These were mostly limited to anti‐inflammatory, antioxidant, and antiapoptotic effects and the regulation of NF‐κB and nuclear factor erythroid 2‐related factor 2 (NRF2) transcription factors, which are involved in regulating cell growth, differentiation, inflammation, antioxidant activity, and apoptosis (Xie et al. 2021, 2020). In this study, we used multiomics techniques to identify changes in metabolites and proteins in septic mice treated with H_2_ and analyzed data from integrated metabolomics and phosphoproteomics databases to determine the functions and signaling pathways of differential proteins and metabolites to explore for the first time the potential mechanisms underlying hydrogen therapy. These findings suggested that glycerophospholipid metabolism was significantly altered in mice in the SAE + H_2_ group compared with mice in the SAE group. Lipids have many critical functions in cellular physiology. Our combined analyses revealed that the levels of choline‐phosphate cytidylyltransferase A (Pcyt1α)/CTP/phosphocholine cytidylyltransferase (CCTα) and DGKζ, two rate‐limiting enzymes of glycerophospholipid metabolism, were significantly downregulated in mice in the SAE + H_2_ group compared to mice in the SAE group. Besides, hydrogen regulates the expression of DGKζ and balancing the DAG and PA signaling pathways in H_2_‐treated septic mice. Further investigation indicated that downregulated DGKζ interacted with PTEN and alleviated brain damage in septic mice after the hydrogen inhalation.

As a vital enzyme in PtdCho synthesis, CCT is indispensable for the regulation of PtdCho homeostasis in mammalian cells. CCT has already been investigated in a variety of diseases, including neurological disorders such as addiction and ischaemia, and alterations in CCT activity related to lipid dysregulation have been shown to result in several disorders of the nervous system (Pati et al. 2019). Pcyt1α/CCTα is located in the nucleus and closely related to cell proliferation, apoptosis resistance, and PtdCho synthesis (Arsenault et al. 2013). Therefore, Pcyt1α/CCTα has been regarded as a novel biomarker for the diagnosis of some cancers. In our study, we found that Pcyt1α/CCTα was downregulated in septic mice treated with hydrogen compared with those without treatment and that the positive effects of hydrogen on septic mice might be associated with Pcyt1α/CCTα activity.

DGKs, as members of the DGK family, are located predominantly in the nucleus and regulate transcription factors during stress responses (Tanaka et al. 2021). In addition, dysfunction of DGKs, which regulate the balance of DAG and PA, might contribute to a variety of diseases, including biodisorders and diabetes (Kakefuda et al. 2010). DGK lies at a crossroads because it controls a plethora of processes for cellular homeostasis. Recently, because of their key role in lipid metabolism, DGKs have been regarded as potential therapeutic targets for cancer progression; for example, the regulation of mTORC1 affects colon cancer survival (Torres‐Ayuso et al. 2015), and the inhibition of cell proliferation and survival in human gliomas is associated with the downregulation of DGKζ (Diao et al. 2016). The DGKζ‐interacting multiprotein complex plays a vital role in regulating p53 and NF‐κB, which are recognized as the major stress responders to p53 degradation and have an inhibitory effect on IκB degradation in the cytoplasm that is independent of catalytic activity (Taniguchi and Karin 2018). Moreover, a recent study revealed that the potential relationship between the downregulation of DGKζ and cervical cancer cell proliferation was related to the ability of DGKζ to facilitate cell apoptosis and cell cycle arrest via the PI3K/Akt and TAK1/NF‐κB signaling pathways (K. Liu et al. 2021). In individuals with sepsis, the homeostasis of T cells and their subsets is disrupted by a variety of mechanisms and different pathogenic factors, and a reduction in the loss of immune cells, the promotion of their activation, and the recovery of their quantity and quality could alleviate sepsis‐induced damage (Hohlstein et al. 2019). Zhong et al. (2003) suggested that DGKζ‐null T cells exhibit TCR‐induced hyperresponsiveness with enhanced activation of the Ras‐ERK cascade, activation markers, cell proliferation, and antiviral immune responses and that DGKζ negatively regulates T‐cell activation and TCR signalling. PTEN plays a crucial role in the processes including excitotoxicity, oxidative stress, apoptosis, and neuroinflammation, influencing key signaling pathways such as PI3K/Akt, MAPK/ERK, and Wnt/β‐catenin (Endicott and Miller 2024; Tufail 2025). Furthermore, PTEN can adjust the activities of PLD and PLC by regulating PIP3, which affects the DGKζ by the regulation of DAG (Ubeysinghe et al. 2023; Kanemaru and Nakamura 2023; Tariq and Luikart 2021; Naik 2010). And our study revealed that the expression of DGKζ was increased in SAE mice but was decreased after the hydrogen treatment, and the regulation of DGKζ might be correlated with the PTEN.

All in all, dysregulated glycerophospholipid metabolism is implicated in SAE, and hydrogen gas alleviates SAE in septic mice by downregulating DGKζ and CCTα activation, which might open a new therapeutic method for modifying SAE. However, we elucidated that the DGKζ of the glycerophospholipid metabolism was a key finding in the septic mice after the hydrogen treatment, although we do not completely explain their underlying mechanisms. Second, we found the interactions between DGKζ and PTEN in SAE mice, and the specific mechanism in the interactions after the hydrogen intervention need our future study to investigate.

## 5 Conclusion

In summary, we used metabolomics and phosphoproteomics techniques to demonstrate that changes in lipids and lipid metabolism (especially glycerophospholipid metabolism) in mice in the SAE + H_2_ group were related to the therapeutic mechanism of hydrogen, and downregulation of the two enzymes Pcyt1α/CCTα and DGKζ, which negatively regulate glycerophospholipid metabolism, preliminarily predicted the molecular mechanisms underlying hydrogen treatment in mice with SAE. We further verified that the low expression of DGKζ regulated by H_2_ could alleviate brain damage in septic mice, which may be related to the regulation of the balance between the DAG and PA. Besides, it might be related to the correlation of PTEN. Our understandings described herein and their underlying mechanisms are far from clear. The exact mechanism underlying the associations between these metabolic alterations and sepsis needs to be validated by subsequent studies.

## Author Contributions

**Yuanyuan Bai**: conceptualization, investigation, writing–review and editing, writing–original draft, methodology. **Zeyu Li**: writing–review and editing, writing–original draft, methodology. **Donglai Yan**: software, validation. **Yi Jiang**: methodology, data curation, formal analysis. **Beibei Dong**: formal analysis, project administration. **Yonghao Yu**: funding acquisition, visualization, supervision, resources.

## Ethics Statement

The mice in this study were provided by the Laboratory Animal Center of the Military Medical Science Academy (Beijing, China). The animal experiments received permission from the Animal Experimental Ethics Committee of Tianjin Medical University General Hospital (Tianjin, China) and were performed in accordance with the National Institutes of Health Guide for Care and Use of Laboratory Animals.

## Conflicts of Interest

The authors declare no conflicts of interest.

## Consent

The authors have nothing to report.

## Peer Review

The peer review history for this article is available at https://publons.com/publon/10.1002/brb3.70761.

## Supporting information




**Supplementary Materials**: brb370761‐sup‐0001‐SupMat.docx

## Data Availability

The data used to support the findings of this study are available from the corresponding author upon request.
