# Supplementary material for: DGKζ in Glycerophospholipid Metabolism Regulates the DAG and PA Balance and Interacts With PTEN to Alleviate Brain Damage in Septic Mice With Hydrogen Inhalation: A Comparative Metabolomic and Phosphoproteomic Analysis
Source: Brain Behav. 2025 Aug 12;15(8):e70761. doi: 10.1002/brb3.70761 (PMC12340545; doi:10.1002/brb3.70761)
Supplement: Supplementary file 1 — Supplementary Materials: brb370761‐sup‐0001‐SupMat.docx [file BRB3-15-e70761-s001.docx]

# Supplementary Materials for

**DGKζ in glycerophospholipid metabolism regulates the DAG and PA balance and interacts with PTEN to alleviate brain damage in septic mice with hydrogen inhalation: A comparative metabolomic and phosphoproteomic analysis**

Yuanyuan Bai et al.

The supplementary material for this manuscript includes the following:

1. **Behavioural experiment**
   1. **Y-maze test**

The Y-maze was made of arms A, B, and C at 120° angles from each other, and the number of alternations/times that each mouse went into all three arms in a row without visiting one arm twice (e.g., mode ABC, BCA, or CAB) was recorded. Each mouse was put into the centre maze and allowed to freely walk in all arms for 10 min. An ANY-maze video tracking system (Stoelting, USA) was used to record the number of alternations and line crossings, and we subsequently analysed the activity of the mice to assess the percentage of alternations.

- 1. **Contextual fear conditioning test**

Contextual fear conditioning test: This test is widely applied for evaluating memory function[18, 19] and consists of three stages: habituation, training, and testing. In the habituation phase, the mice were put into the training context with free movement for 10 min. In the training phase, the mice were placed in a fear chamber one day before modelling, acclimated for 2 min, and then given 20 s of a single-frequency sound signal (70 dB) coterminating with a foot shock (0.70 mA, 2 s). After an interval of 25 s, the auditory stimulus was played for another 60 s, coterminating with a second foot shock, marking the end of one full cycle of training (105 s). Six cycles of training were administered. The mice displayed panic, escape attempts, or rigidity when they heard the sound signal (no motor behaviour other than breathing) and squealing, jumping and escape when they were shocked, indicating the formation of fear memory. A sham or CLP model was established the day after fear memory formation. Contextual fear memory tests were administered at 1, 2, 3, 5 and 7 d after sham surgery or CLP. During the test period, the mice were placed into the fear box, which was the same as the environment during the training period except that they were not given the sound signal or electrical stimulation and were allowed to move freely for 300 s. An ANY-maze video analysis system was used to record the time of rigidity in each group during the training period and the test period, and the percentage of the time of rigidity was calculated. The mice were regarded as freezing if there was no movement for 2 s (freezing time / 300 s × 100% = freezing time ratio).

1. **Supplementary methods for metabolomic and phosphoproteomic analyses**

**2.1. Sample collection**

Whole brain tissues from 24 mice from the four treatment groups were flash frozen in liquid nitrogen and subsequently cut and placed in Eppendorf tubes. The samples were homogenized in a homogenizer with 0.2 ml of H_2_O and five ceramic beads. Then, 0.8 ml of methanol/acetonitrile (1:1, v/v) was added to the homogenized solution for metabolite extraction. The mixture was subsequently centrifuged for 15 min (14000 × g, 4 °C). The supernatant was dried in a vacuum centrifuge. The samples were redissolved in 0.1 ml of acetonitrile/water (1:1, v/v) for LC‒MS analysis. Quality control (QC) samples were generated by pooling 10 μl of each sample and analysed regularly after every 5 samples to measure instrument stability and repeatability.

**2.2****. LC‒MS/MS analysis**

LC‒MS/MS analysis was performed using a UHPLC instrument (1290 Infinity LC, Agilent Technologies) coupled to a quadrupole time‒of-flight mass spectrometer (AB SCIEX TripleTOF 6600) at Shanghai Applied Protein Technology Co., Ltd. For HILIC separation, the samples were separated using a 2.1 × 100 mm ACQUITY UPLC BEH 1.7-µm column (Waters, Ireland). In both positive and negative electrospray ionization (ESI) modes, the mobile phase contained 25 mM ammonium acetate and 25 mM ammonium hydroxide in water (mobile phase A) and acetonitrile (mobile phase B). The gradient was 85% B for 1 min, which was linearly reduced to 65% in 11 min, decreased to 40% in 0.1 min, maintained for 4 min, and then increased to 85% in 0.1 min, with a 5 min re-equilibration period. For RPLC separation, a 2.1 mm × 100 mm ACQUITY UPLC HSS T3 1.8-µm column (Waters, Ireland) was used. In positive ESI mode, the mobile phase consisted of water with 0.1% formic acid (mobile phase A) and acetonitrile with 0.1% formic acid (mobile phase B); in negative ESI mode, the mobile phase consisted of 0.5 mM ammonium fluoride in water (mobile phase A) and acetonitrile (mobile phase B). The mobile phase gradient was 1% B for 1.5 min, linearly increased to 99% in 11.5 min and maintained for 3.5 min. Subsequently, B was reduced to 1% in 0.1 min, with a 3.4-min re-equilibration period. The flow rate was 0.3 ml/min, and the column temperature was held constant at 25 °C. A 2-µl aliquot of each sample was injected. The ESI source parameters were set as follows: ion source gas 1 (Gas 1), 60; ion source gas 2 (Gas 2), 60; curtain gas (CUR), 30; source temperature, 600 °C; and ion spray voltage floating (ISVF), ± 5500 V. In MS-only acquisition, the instrument was set to acquire data over the m/z range of 60-1000, and the accumulation time for the TOF MS scan was set at 0.20 s/spectra. For automatic MS/MS acquisition, the instrument was set to acquire data over the m/z range of 25-1000, and the accumulation time for the product ion scan was set at 0.05 s/spectra. The product ion scan was acquired in information-dependent acquisition (IDA) mode with high-sensitivity mode selected. The parameters were set as follows: collision energy (CE), 35 V ± 15 eV; declustering potential (DP), 60 V (+) and −60 V (−); exclusion of isotopes within 4 Da; and number of candidate ions for monitoring each cycle, 10.

**2.3. Data processing**

The raw MS data were converted into mzXML files via ProteoWizard msConvert before being imported into free XCMS software. The Collection of Algorithms MEtabolite pRofile Annotation (CAMERA) algorithm set was used for the annotation of the isotopes and the adducts. Among the extracted ion features, only those with at least one set of variables with nonzero measurements exceeding 50% were retained. Metabolite identification was performed by comparing the accuracy of the m/z values (<10 ppm) and MS/MS profiles with an in-house database established with existing confirmatory standards.

**2.4. Statistical analysis**

After sum-normalization, the processed data were analyzed by R package (ropls), where it was subjected to multivariate data analysis, including Pareto-scaled principal component analysis (PCA) and orthogonal partial least-squares discriminant analysis (OPLS-DA). The 7-fold cross-validation and response permutation testing were used to evaluate the robustness of the model. The variable importance in the projection (VIP) value of each variable in the OPLS-DA model was calculated to indicate its contribution to the classification. Student’s t test was applied to determine the significance of differences between two groups of independent samples. VIP > 1 and p value < 0.05 were used to screen significant changed metabolites. Pearson’s correlation analysis was performed to determine the correlation between two variables.
